# Hematological Parameter-Derived Inflammatory Scores in Non-Pancreatic Hyperlipasemia (NPHL)—The Prognosis Lies in the Blood

**DOI:** 10.3390/biomedicines13071719

**Published:** 2025-07-14

**Authors:** Krisztina Eszter Feher, David Tornai, Maria Papp

**Affiliations:** 1 Division of Gastroenterology, Department of Internal Medicine, Faculty of Medicine, University of Debrecen, 4032 Debrecen, Hungary; feher.krisztina.eszter@med.unideb.hu (K.E.F.);; 2 Kalman Laki Doctoral School of Biomedical and Clinical Sciences, Faculty of Medicine, University of Debrecen, 4032 Debrecen, Hungary

**Keywords:** elevated lipase, inflammation, bacterial infection, sepsis, viral infection, COVID-19, SARS-CoV-2, mortality, neutrophil-to-lymphocyte ratio

## Abstract

**Background/Objectives:** Non-pancreatic hyperlipasemia (NPHL) is associated with high in-hospital mortality, with sepsis being one of the most common etiologies. The prognostic value of hematological parameter-derived inflammatory scores has not been extensively studied in NPHL to date. **Methods:** The prognostic value of eight inflammatory scores for in-hospital mortality was assessed in a total of 545 NPHL patients from two hospitalized patient cohorts (COVID-19 [n = 144] and non-COVID-19 [n = 401], the latter stratified as bacterial sepsis [n = 111] and absence of systemic infection [n = 290]). We assessed the neutrophil-to-lymphocyte ratio (NLR), derived NLR (dNLR), neutrophil-to-lymphocyte and platelet ratio (N/(LP)), platelet-to-lymphocyte ratio (PLR), lymphocyte-to-monocyte ratio (LMR), aggregate index of systemic inflammation (AISI), systemic inflammation index (SII), and systemic inflammation response index (SIRI), comparing their prognostic value among etiological groups. **Results:** Patients with bacterial sepsis were older, had more comorbidities, and experienced worse outcomes, including longer hospitalization (median: 15, 7, and 11 days; *p* < 0.001), higher ICU admission rates (75.7%, 33.8%, and 47.9%, *p* < 0.001), and increased mortality (45.0%, 13.8%, and 38.2%, *p* < 0.001), compared to those without systemic infection or with COVID-19-induced NPHL. Overall, NLR, dNLR, and N/(LP) were the most accurate predictors of in-hospital mortality at admission (AUROC: non-infection: 0.747; 0.737; 0.772; COVID-19: 0.810; 0.789; 0.773, respectively). The accuracy of NLR decreased in bacterial sepsis, and only N/(LP) and PLR remained associated with in-hospital mortality (AUROC: 0.653 and 0.616, respectively). **Conclusions:** The prognostic performance of hematological parameter-derived inflammatory scores in NPHL is etiology-dependent. NLR is the most accurate prognostic tool for mortality in the absence of bacterial sepsis, while N/(LP) is the best score in sepsis-induced NPHL.

## 1. Introduction

Serum lipase elevation is a well-established serological hallmark for the diagnosis of acute pancreatitis (AP) [1]. However, elevated lipase levels can also occur in a variety of other clinical conditions unrelated to direct pancreatic inflammation, including diseases associated with lipase leakage due to the inflammation of peripancreatic abdominal organs or impaired clearance resulting from reduced renal function and/or hepatic metabolism [2,3]. The term non-pancreatic hyperlipasemia (NPHL) refers to elevated serum lipase levels in the absence of radiological signs of pancreatitis and characteristic abdominal pain [1]. Hameed et al. [3] were the first to systematically summarize the alternative etiologies responsible for hyperlipasemia without pancreatitis. In a recent publication by our group, we identified over 20 different causes of NPHL, of which bacterial sepsis was the most frequent etiology, accounting for 27.7% of cases. Other contributing conditions included acute kidney injury (AKI), malignancies (pancreatic, non-pancreatic, and hematological), hepatobiliary disorders (e.g., cholangitis, cholecystitis, liver disease), gastrointestinal pathologies (e.g., bowel obstruction, ischemia, perforation, bleeding, and inflammatory bowel disease), metabolic disturbances such as diabetic ketoacidosis, cardiovascular and neurosurgical conditions, post-ERCP states, and drug-induced lipase elevation. Notably, in our Hungarian study population, patients with NPHL experienced four-fold higher in-hospital mortality rates than those diagnosed with AP (22.4% vs. 5.1%, *p* < 0.001) [4].

Sepsis represents a major underlying cause of NPHL, as a key driver of systemic inflammation. It remains a major global healthcare burden, responsible for an estimated 11 million deaths annually according to the World Health Organization (WHO) 2020 report [5]. The pathophysiology of sepsis involves an excessive host response to infection, frequently initiating a cascade of inflammatory events, and might lead to multiorgan dysfunction. Non-infectious agents can also contribute to systemic inflammation, eliciting a complex immune response involving both innate and adaptive mechanisms [6]. While conventional biomarkers such as C-reactive protein (CRP) and procalcitonin (PCT) have long been used in the clinical management of sepsis, increasing attention has been directed toward hematological inflammation-based indices. These blood count-derived markers reflect different aspects of the inflammatory response and, in several clinical settings, have demonstrated superior prognostic accuracy for mortality prediction [7]. Among these, the neutrophil-to-lymphocyte ratio (NLR), introduced by Zahorec in 2001 [8], has gained prominence due to its simplicity and accessibility, reflecting the balance between innate and adaptive immune responses.

More recently, attention has turned to viral infections, including SARS-CoV-2, as potential contributors to hyperlipasemia and systemic inflammation [9]. Although SARS-CoV-2 primarily affects the respiratory tract, it also has recognized extrapulmonary manifestations, including gastrointestinal and pancreatic involvement [10,11]. The virus gains cellular entry via the angiotensin-converting enzyme (ACE)-2 receptor, which is expressed not only in the respiratory epithelium but also in other tissues, such as the gastrointestinal tract, liver, and pancreas [12,13]. Liu et al. [14] demonstrated that ACE-2 receptor expression is even higher in pancreatic tissue than in the respiratory epithelium. Pancreatic involvement may lead to interstitial lipase leakage and adipose tissue lipolysis, promoting fatty acid accumulation and mitochondrial injury. These processes contribute to cytokine storm development, multiorgan failure, and ultimately, COVID-19-related mortality [15]. However, the prognostic relevance of inflammatory indices in COVID-19-associated NPHL has not been specifically evaluated, necessitating dedicated investigation.

In our previous observational study, we demonstrated that NLR was an excellent prognostic marker in NPHL, identifying patients at higher risk of in-hospital mortality with an optimal cut-off value of >10.37 (OR: 3.71, 95% CI: 2.006–6.863; *p* < 0.001). Furthermore, the prognostic ability of NLR in this setting surpassed that of traditional inflammatory markers such as C-reactive protein (CRP) and procalcitonin (PCT) or pancreas-related laboratory parameters such as lipase [4].

However, to date, several widely used hematological parameter-derived inflammatory indices—including the derived neutrophil-to-lymphocyte ratio (dNLR) [16], the neutrophil-to-lymphocyte and platelet ratio (N/(LP)) [17], the platelet-to-lymphocyte ratio (PLR) [18], and the lymphocyte-to-monocyte ratio (LMR) [19], as well as composite scores such as the aggregate index of systemic inflammation (AISI) [20], the systemic inflammation index (SII) [21], and the systemic inflammation response index (SIRI) [22]—have not been evaluated in the prognosis of NPHL patients.

These indices emerged from diverse clinical backgrounds, each aiming to quantify different facets of systemic inflammation and immune imbalance. The derived neutrophil-to-lymphocyte ratio [16] gained initial recognition in colorectal cancer, where it independently predicted overall survival; N/(LP) [17] was proposed in the setting of acute myocardial infarction, with elevated values linked to both short- and long-term mortality. The PLR [18] entered clinical use as a marker of inflammation in end-stage renal disease and has since been applied across cardiovascular and oncological [23] contexts, where its prognostic performance has varied depending on disease type and inflammatory burden. In infectious disease, LMR [19] was introduced as a diagnostic aid in influenza, where lower ratios were associated with immune suppression and more severe clinical presentation; later studies extended its use to cancer [24] and cerebrovascular [25] conditions. The aggregate index of systemic inflammation [20] was developed in chronic obstructive pulmonary disease, where it demonstrated associations with reduced lung function and increased mortality. Meanwhile, the systemic immune inflammation index [21] found utility in hepatocellular carcinoma, showing robust predictive value for both overall and disease-free survival, and has since been adopted in a range of malignancies and inflammatory disorders. Finally, the SIRI [22] was initially applied in pancreatic and lung cancer, where elevated levels were linked to aggressive disease behavior and poorer outcomes, and it has since gained attention in infectious [26] and thromboinflammatory diseases [27] as well. The first clinical applications and the corresponding references of each index are summarized in Table 1.

Identifying the most reliable, readily available tools for the prediction of adverse outcomes in NPHL of various etiologies is crucial to assist clinical decision-making and optimize patient management. Therefore, the primary objective of the present study was to comprehensively evaluate the prognostic performance of a wide range of hematologic parameter-derived inflammatory scoring systems parallelly for adverse outcome in hospitalized patients with NPHL.

Specifically, we aimed to (a). identify the most accurate inflammatory score for in-hospital mortality prediction in NPHL; (b). investigate how the presence of bacterial sepsis influences the overall prognostic value of the indices; and (c). compare the prognostic performance of the various scores in COVID-19-associated NPHL and non-COVID-19-associated NPHL cases. These aims are expected to support the risk stratification of patients with NPHL in various clinical contexts.

## 2. Materials and Methods

This study involved two distinct hospitalized patient cohorts. The primary cohort consisted of adult patients prospectively enrolled at the Clinical Center of the University of Debrecen between June 2017 and December 2019 [4]. Patients were included if they exhibited serum lipase levels ≥3 times the upper limit of normal (ULN) upon admission. Patients diagnosed with acute pancreatitis (AP; n = 392), according to the American Gastroenterological Association (AGA) [1] guideline, or chronic pancreatitis (n = 15) were excluded [28]. The final study population comprised 401 patients with non-pancreatic hyperlipasemia (NPHL), defined by elevated serum lipase ≥3× ULN in the absence of characteristic abdominal pain and without radiological signs of pancreatitis on computed tomography (CT), magnetic resonance imaging (MRI), or ultrasonography. These non-COVID-19 NPHL patients were further divided into septic and non-septic subgroups based on their underlying etiology. The secondary cohort served as a model to investigate NPHL with severe viral infection. This retrospective cohort included patients hospitalized due to COVID-19 infection at the Clinical Center of the University of Debrecen from March 2020 to March 2023. The clinical and laboratory data of 15,564 consecutive COVID-19 inpatients were screened. Lipase measurements were available for 3.391 patients at admission, among whom 192 (5.7%) presented with serum lipase levels ≥3× ULN. After the exclusion of 48 patients (1.4%) with AP, the control group comprised 144 (4.2%) patients with COVID-19-associated NPHL.

### 2.1. Data Collection

Demographic data included patients’ age and gender. Clinical data comprised the Charlson Comorbidity Index (CCI), which accounts for comorbidities such as cardiovascular and cerebrovascular diseases, respiratory disorders, connective tissue diseases, peptic ulcer, liver and renal disorders, diabetes mellitus, solid tumors, leukemia, lymphoma, and acquired immune deficiency syndrome [29]. The presence of bacterial sepsis was also recorded. The following clinical outcomes were assessed: length of hospitalization, intensive care unit (ICU) admission, and in-hospital mortality.

Biochemical parameters collected at admission included serum sodium, albumin, blood urea nitrogen (BUN), creatinine, amylase, and lipase levels, as well as inflammatory markers such as C-reactive protein (CRP) and procalcitonin (PCT). Hematological parameters consisted of white blood cell (WBC) count and differential counts of neutrophils, lymphocytes, monocytes, and platelets.

Eight different hematological parameter-derived inflammatory scores were calculated at admission. Table 1 summarizes the formulas used for calculating the inflammatory indices based on routinely available hematological parameters, including neutrophil, lymphocyte, monocyte, platelet, and total white blood cell counts, according to guidance in the literature.

All laboratory parameters were measured using standard automated methods at the Laboratory Medicine Institute of Clinical Center during the routine patient care. The diagnosis of sepsis was established by the treating physician based on the Sepsis-2 consensus definition [30].

### 2.2. Statistical Analysis

Categorical variables were summarized as n (%) and compared using the χ^2^ test or Fisher’s exact test, as appropriate. Continuous variables were summarized as medians [interquartile range (IQR)] and compared using the Mann–Whitney *U* test. The relationship between continuous variables was assessed using non-parametric Spearman’s correlation. The association between variables and outcomes was estimated using receiver operating curve (ROC) analysis by assessing sensitivity vs. 1-specificity. The area under the receiver operating characteristic (AUROC) and the corresponding 95% confidence intervals (CIs) were calculated. The Youden index was chosen, calculated as the maximum (sensitivity + specificity − 1) value, to estimate the best discriminate thresholds. Sensitivity and specificity values were given to characterize the predictive power of variables. Binary logistic regression was used to assess the relationship between inflammatory scores and in-hospital mortality. Independent predictors were assessed by multivariable logistic regression using the enter method. Associations are presented as odds ratios (ORs) with 95% CIs. A 2-sided probability value < 0.05 was considered significant. For statistical analysis, the SPSS 29.0 (SPSS Inc., Chicago, IL, USA) program was used. The statistical methods used in this study were reviewed by Professor Elek Dinya, PhD, DSc, Semmelweis University, Institute of Health Informatics, Budapest, Hungary.

### 2.3. Ethical Permission

This study was conducted in accordance with the Declaration of Helsinki and reviewed and approved by the Hungarian National Review Board (ETT TUKEB) and the Institutional Review Board of the University of Debrecen (RKEB) (305/2014, 30595-1/2014 EKU, 55961-2/2016/EKU, 5753-2/2018 EKU, 6136-2022). Study management strictly followed the Ethical Guidelines for Observational Studies.

## 3. Results

### 3.1. Study Population

A total of 545 patients with non-pancreatic hyperlipasemia (NPHL) were included in the study. Of the 401 patients from our previously established cohort, 111 had proved bacterial sepsis [4]. Additionally, 144 COVID-19-associated NPHL patients were enrolled to control for severe viral infection.

Table 2 summarizes the demographic characteristics, comorbidities, clinical outcomes, laboratory parameters, and inflammatory indices of the study groups, as follows: NPHL without systemic infection, NPHL with severe bacterial infection, and COVID-19-associated NPHL. To provide further clinical context, individual underlying conditions and causes of death for patients in the non-infectious NPHL subgroup are presented in Table S1 (Supplementary Materials). In the overall study population, males predominated (55.4%), with a median age of 66 years (IQR: 56–76), and 92.5% of the patients presented with at least one comorbidity. Patients in the bacterial sepsis group were significantly older and had a greater comorbidity burden, as reflected by higher Charlson Comorbidity Index (CCI) scores compared to those without systemic infection [median (IQR): 6 (4–9) vs. 5 (3–8); *p* = 0.001]. In contrast, patients with COVID-19-associated NPHL had comparable age but significantly lower CCI values [median (IQR): 3 (2–5); *p* < 0.001].

Both the bacterial and viral infection groups exhibited a more severe clinical course compared to patients without systemic infection, demonstrated by prolonged hospital stays (median: 15 and 11 vs. 7 days; overall *p* < 0.001), increased rates of intensive care unit (ICU) admission (75.7% and 47.9% vs. 33.8%, overall *p* < 0.001), and higher in-hospital mortality rates (45.0% and 38.2% vs. 13.8%, overall *p* < 0.001).

Regarding laboratory parameters, serum lipase and amylase levels were significantly elevated in the bacterial sepsis group compared to the non-infectious and viral infection groups [lipase median (IQR): 425 (263–651) vs. 266 (231–435) vs. 300.5 (243–556) U/L, *p* < 0.001 for both comparisons; amylase median (IQR): 221 (132–344) vs. 165 (110–229) vs. 167 (102–241) U/L, *p* < 0.001 for both]. Furthermore, markers of disease severity, including serum albumin and renal function parameters, were more profoundly altered in patients with bacterial sepsis. Similarly, inflammatory markers (CRP, PCT, WBC, and neutrophil granulocyte count) showed markedly higher values in the bacterial sepsis group.

Finally, values of all calculated hematological parameter-derived inflammatory scores were significantly elevated in both the bacterial sepsis and viral infection groups compared to patients without systemic infection, with the highest values consistently observed in the bacterial sepsis group.

### 3.2. Correlation Analysis of Inflammatory Markers

**Figure 1:**
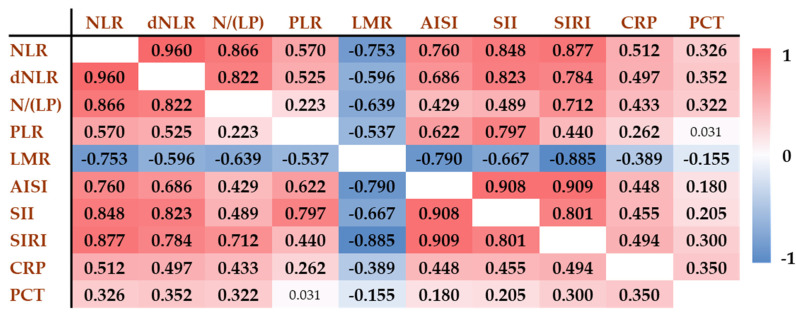
Correlation of inflammatory biomarkers and hematological parameter-derived inflammatory scores in the original non-COVID-19 population (n = 401). Positive correlations are shown in red, while negative correlations are indicated in blue. The darker the color, the stronger the correlation. Numbers refer to the correlation coefficients (r values). The r values associated with statistically significant *p* values are indicated in bold. NLR: neutrophil-to-lymphocyte ratio; dNLR: derived neutrophil-to-lymphocyte ratio; N/(LP): neutrophil-to lymphocyte and platelet ratio; PLR: platelet-to-lymphocyte ratio; LMR: lymphocyte-to monocyte ratio; AISI: aggregate index of systemic inflammation; SII: systemic inflammation index; SIRI: systemic inflammation response index; CRP: C-reactive protein; PCT: procalcitonin.

The correlation matrix (Figure 1) demonstrates the interrelationships among various inflammatory markers and hematological parameter-derived inflammatory scores across the original non-COVID-19 study population. Strong positive correlations were observed between NLR and SIRI (r = 0.877), SIRI and AISI (r = 0.909), as well as between SII and AISI (r = 0.908). These findings reflect the shared components of these composite indices and their similar behavior in systemic inflammation.

In contrast, LMR showed strong inverse correlations with several markers, most notably with SIRI (r = −0.885), AISI (r = −0.790), and SII (r = −0.667), indicating its opposing trend in inflammatory states. PLR showed generally weaker correlations with the other indices and demonstrated practically no correlation with PCT (r = 0.031) and a weak correlation with CRP (r = 0.262).

These results highlight that while several indices, such as SIRI, AISI, and NLR, move in parallel and are strongly interrelated, other markers, like LMR, provide distinct and potentially complementary information in the assessment of systemic inflammation.

### 3.3. Performance of Hematological Parameter-Derived Inflammatory Scores in Predicting In-Hospital Mortality in the Original Non-COVID-19 Study Population

The diagnostic and prognostic potential of various inflammatory scores was assessed via a comparison between survivors (n = 311) and non-survivors (n = 90) and using receiver operating characteristic (ROC) curve analysis in the original non-COVID-19 study population (Table 3).

Most markers showed significantly higher median values in non-survivors compared to survivors (all *p* < 0.001), except for PLR, which did not differ significantly between the groups (*p* = 0.605). The NLR demonstrated good discriminatory ability, with an AUROC of 0.747 (95% CI: 0.691–0.803) and a cut-off value >10.37, yielding a sensitivity of 65.17% and a specificity of 78.64%. The N/(LP) ratio showed the highest prognostic performance, with an AUROC of 0.772 (95% CI: 0.717–0.827) and a cut-off >0.03, providing 78.65% sensitivity and 64.72% specificity. Similarly, dNLR and SIRI had robust AUROC values of 0.737 and 0.722, respectively. Conversely, LMR was inversely associated with mortality (AUROC: 0.664), and AISI and SII also showed moderate predictive value (AUROCs: 0.630 and 0.650, respectively). PLR had the weakest performance (AUROC: 0.518), suggesting limited utility for mortality risk stratification in this population. These findings highlight the potential clinical value of composite inflammatory indices, particularly N/(LP), NLR, and SIRI, as predictive tools in the identification of patients at elevated risk of in-hospital mortality.

Univariable logistic regression analysis was performed to assess the association between inflammatory biomarkers and in-hospital mortality following dichotomization based on optimal cut-off values (binary variables) (Table 4). NLR and N/(LP) exhibited the strongest predictive power, with ORs of 6.889 and 6.480, respectively. These were followed by SIRI (OR = 5.119) and dNLR (OR = 4.846). These findings confirm the robustness of NLR, dNLR, and N/(LP) as reliable predictors of in-hospital mortality.

To evaluate the prognostic value of N/(LP) in a broader clinical context, we reconstructed the multivariable model from our previous publication by replacing NLR with N/(LP) while retaining all other clinical and laboratory variables (Table 5). N/(LP) remained an independent predictor of in-hospital mortality with a slightly higher odds ratio compared to the original model using NLR (OR: 3.98 vs. 3.71). Other independent predictors included age, presence of sepsis, elevated amylase, and hypoalbuminemia.

To investigate subgroup-specific performance, we analyzed the prognostic value of hematological parameter-derived inflammatory scores separately in patients without systemic infection (n = 290; Table 6). The results were largely consistent with those observed in the overall cohort (Table 3). NLR, dNLR, and N/(LP) demonstrated the highest AUROC values (0.7805, 0.7506, and 0.773, respectively) and were significant predictors of in-hospital mortality in univariable logistic regression analyses (OR (CI): 10.71 (4.06–28.29); 6.65 (3.23–13.71); 6.09 (3.01–12.34); 5.73 (2.79–11.76), respectively). In contrast, PLR showed poor prognostic performance and was not statistically significant in our analysis. AISI, SII, and LMR demonstrated moderate predictive ability, with the latter being inversely associated with mortality.

In the subgroup of NPHL patients with sepsis (n = 111) (Table 7), the prognostic performance of hematological parameter-derived inflammatory scores was limited overall. Most scores demonstrated poor discriminatory ability, with AUROCs below 0.60. The NLR and dNLR were elevated in non-survivors compared to survivors, but neither reached statistical significance (AUROC 0.568 and 0.563, respectively; *p* > 0.2 for both). Notably, two indices incorporating platelet count—N/(LP) and PLR—retained significant associations with mortality. N/(LP) showed the best overall performance, with an AUROC of 0.653 (95% CI: 0.549–0.757, *p* = 0.006) and a significantly increased odds of mortality above the best discriminatory cut-off value (OR (CI): 3.80 (1.67–8.60)). PLR also reached statistical significance (AUROC: 0.616, *p* = 0.038), although not elevated but decreased levels (≤171.15) were predictive of mortality (OR (CI): 2.30 (1.06–5.02)). Other indices, including the LMR, AISI, SII, and SIRI, did not show significant predictive value in this subgroup.

In the COVID-19-associated NPHL control group (n = 144), most inflammatory indices demonstrated strong discriminatory ability for in-hospital mortality. The NLR showed the highest predictive performance, with an AUROC of 0.810 (95% CI: 0.736–0.884, *p* < 0.004), and was strongly associated with mortality in univariable logistic regression at the cut-off of >10.65 (OR (CI): 15.86 (6.79–37.05)). Similarly, the dNLR, the N/(LP), and the SII all reached AUROC values above 0.76 and were predictive of mortality in logistic regression (OR (CI): 13.37 (5.85–30.54); 5.17 (2.46–10.84); 5.91 (2.78–12.53), respectively). The PLR was also markedly elevated in non-survivors (median: 301.3 vs. 158.9), with an AUROC of 0.726. The LMR and AISI were inversely associated with mortality and showed modest AUROCs (0.618 and 0.608, respectively (Table 8).

## 4. Discussion

In our previously published prospective study on non-pancreatic hyperlipasemia (NPHL; n = 401) [4], we demonstrated that NPHL is not merely a laboratory abnormality but a clinically meaningful condition associated with significantly higher in-hospital mortality than acute pancreatitis (AP). One major contributor to this excess mortality was the frequent presence of sepsis, affecting nearly one-third of patients. Despite the routine use of biochemical markers such as CRP, PCT, and serum lipase, none proved to be reliable predictors of outcome in this patient population.

Given these limitations, we sought to identify accessible and more effective prognostic markers. We selected the NLR for further analysis, as it is among the most extensively studied systemic inflammatory indices. First described by Zahorec [8], NLR reflects the balance between innate and adaptive immunity and has demonstrated strong prognostic value in numerous clinical settings. In our earlier study, NLR outperformed traditional markers, and we found that an NLR > 10.37 was independently associated with nearly fourfold increased odds of in-hospital death of NPHL patients [4].

Building on these observations, we investigated whether other composite hematological parameter-derived inflammatory indices—such as dNLR, N/(LP), PLR, LMR, SII, AISI, and SIRI—might provide superior prognostic value. These markers integrate various leukocyte subtypes and platelets and may better capture the complexity of immune dysregulation in critical illness [31,32].

To understand the context-dependent performance of these indices, we stratified the NPHL population into three etiological subgroups: patients without systemic infection, patients with bacterial sepsis, and patients with severe viral (COVID-19) infection. This distinction was critical, as sepsis is a high-mortality clinical condition where immune exhaustion may alter marker performance. Meanwhile, COVID-19 infection also represents a severe disorder with high mortality [33] in which inflammatory indices have been well-studied—but not specifically in the context of COVID-19-associated NPHL. We were therefore particularly interested in whether the predictive power of these indices differed between non-COVID-19 NPHL subtypes (septic vs. non-infectious) and COVID-19-induced NPHL.

In the group of non-COVID-19 NPHL patients—including both septic and non-infectious etiologies (n = 401)—the N/(LP) index demonstrated the most consistent and robust prognostic performance across multiple analytic approaches. It achieved the highest discriminatory power in ROC analysis, with an AUROC of 0.772 (95% CI: 0.717–0.827), outperforming other markers such as NLR (AUROC = 0.747) and dNLR (AUROC = 0.737). In univariable logistic regression, both N/(LP) and NLR were strongly associated with in-hospital mortality, with odds ratios of 6.480 and 6.889, respectively. Importantly, in the multivariable model that included age, albumin, amylase, and sepsis status, N/(LP) remained a statistically significant independent predictor of in-hospital death. Values above the optimal threshold (>0.03) were associated with an almost four-fold increase in the odds of mortality (OR = 3.980; 95% CI: 2.039–7.768; *p* < 0.001), which was slightly higher than we previously found with NLR (OR: 3.71) [4]. These findings suggest that NLR and N/(LP) provide similar predictive value in non-COVID-19 NPHL.

In the non-infectious subgroup (n = 290), NLR had the highest AUROC (0.7805), followed closely by N/(LP) (0.773). While NLR demonstrated better sensitivity (87.5%), N/(LP) showed superior specificity (80.24%), reinforcing its clinical relevance. Other indices such as dNLR and SIRI also performed well, while PLR failed to reach statistical significance.

Although the diagnostic ability of most hematological parameter-derived inflammatory indices was markedly attenuated in our septic (n = 111) NPHL subgroup—likely due to an extensively elevated inflammatory state that masked the ability of these indices to provide further stratification—the N/(LP) index preserved a statistically significant but moderate prognostic value (AUROC: 0.653, *p* = 0.006, OR: 3.80, *p* = 0.001). These findings are consistent with previous studies reporting on the prognostic utility of N/(LP) in cardiovascular surgery and sepsis-associated acute kidney injury [17,34]. Notably, in our non-COVID-19 population, 74% (n = 82/111) of patients with sepsis had acute kidney injury [4]. Our findings are also in line with the results of Zhang et al. [35], who retrospectively analyzed 195 adult ICU patients with sepsis and reported significantly higher initial N/(LP) values among non-survivors. In their study, N/(LP) demonstrated good discriminatory performance (AUROC = 0.763) and was an independent predictor of 28-day mortality. While the magnitude of predictive accuracy was lower in our cohort, the consistent direction of association reinforces the potential utility of N/(LP) in septic settings. Notably, the prognostic value of N/(LP) in sepsis has also been confirmed in pediatric populations. In a recent study involving 230 critically ill children with sepsis, N/(LP) demonstrated the highest predictive accuracy for in-hospital mortality (AUROC = 0.748), comparable to the Pediatric Critical Illness Score, and was significantly associated with 30-day mortality [36]. Interestingly, a recent large-scale study by Zhang et al. [37], NLR was also identified as an independent predictor of mortality in sepsis. Moreover, when incorporated into a multivariable nomogram model—including Sequential Organ Failure Assessment (SOFA), red cell distribution width (RDW), Charlson Comorbidity Index (CCI), lymphocyte-to-monocyte ratio (LMR), and mean corpuscular volume (MCV)—NLR contributed to an overall model AUC of 0.724, significantly outperforming the SOFA score alone (AUC = 0.585) [37]. Conversely, the NLR demonstrated no association with in-hospital mortality in our septic NPHL population, even in univariable analysis. This finding suggests that the presence of sepsis altered the predictive ability of inflammatory indices in our study population. This may reflect a saturation effect, whereby the intense systemic inflammation in sepsis limits the discriminatory capacity of NLR. In contrast, N/(LP), which also includes platelets, retained prognostic value—likely because it captures additional thromboinflammatory processes relevant to sepsis-associated NPHL.

A critical methodological distinction must be acknowledged; while Zhang et al. applied the Sepsis-3 criteria [38], we employed the Sepsis-2 [30] definition, as it aligned more closely with the pathophysiological characteristics of the hematological inflammatory markers under investigation. Sepsis-3 emphasizes organ dysfunction, whereas Sepsis-2 defines sepsis as the coexistence of SIRS and infection, making it more sensitive to early systemic immune responses—such as neutrophilia, lymphopenia, and dynamic platelet count changes [39]. Given that the prognostic performance of inflammatory indices is influenced by the underlying inflammatory milieu, Sepsis-2 provided a more biologically coherent framework for classifying this patient population. This approach was particularly justified in the context of non-pancreatic hyperlipasemia (NPHL), where atypical immune responses are common and early systemic inflammation is more accurately captured by a SIRS-based definition. 

In COVID-19-associated NPHL (n = 144), the prognostic accuracy of hematological parameter-derived inflammatory indices was markedly improved compared to other etiologies. Most markers, including NLR, dNLR, N/(LP), PLR, and SII, showed strong discriminatory performance, all with AUROC values above 0.72 and significant associations with in-hospital mortality. Among these, the NLR exhibited the best prognostic accuracy (AUROC = 0.810; 95% CI: 0.736–0.884; *p* < 0.004), with an odds ratio of 15.86 (95% CI: 6.79–37.05), indicating a more than fifteen-fold increase in the odds of mortality above the optimal threshold. These findings are in line with prior studies that highlighted the prognostic value of hematological parameter-derived inflammatory markers in COVID-19 [40,41,42] but without subgroup analysis for NPHL. In this regard, our study provides a novel insight. Notably, the AUROC we observed for NLR in this hyperlipasemic subgroup was considerably higher (0.810) than that reported by others. Fois et al. [42] found an AUROC of 0.697 (95% CI: 0.605–0.778; *p* = 0.002) in a general hospitalized COVID-19 population. In a 2024 retrospective study from Mexico evaluating 189 hospitalized COVID-19 patients, an NLR cut-off of 8.6 yielded an AUC of 0.678 (*p* < 0.0001), with 70.4% sensitivity and 51.8% specificity [43]. This study also found elevated PLR, and SII values to be significantly associated with increased mortality. In a large Italian multi-cohort validation study by Colaneri et al. [44], which evaluated NLR and PLR in over 3500 hospitalized COVID-19 patients, NLR demonstrated consistent predictive utility across three different hospitals, with sensitivities ranging from 54% to 67% and specificities between 64% and 76% for mortality prediction, although no AUROC was directly reported. These differences support NPHL as a distinct clinical entity with altered immunological responses requiring a re-evaluation of prognostic approaches. Importantly, according to our unpublished results, COVID-19-associated NPHL patients have an increased mortality rate compared to those with COVID-19 without NPHL (42.1% vs. 26.4% *p* < 0.001).

The consistent prognostic value of the N/(LP) ratio across all three subgroups in our study may be partly attributed to the multifaceted pathophysiological role of platelets. Platelets are not only central to hemostasis but are also potent sources of proinflammatory mediators and bioactive metabolites [45]. Their interaction with leukocytes—first described by Bizzozero [46] in 1882—is a key link between the inflammatory and thrombotic cascades [30]. During early inflammation, platelet counts tend to rise; however, in advanced sepsis, thrombocytopenia often ensues due to peripheral sequestration or increased consumption [47,48]. Beyond infection, platelets have also been implicated in promoting tumor progression through immune evasion mechanisms [49]. These diverse roles support the inclusion of platelet count in composite inflammatory indices such as N/(LP) and SII, potentially enhancing their sensitivity to immune activation, endothelial dysfunction, and coagulopathy—hallmarks of adverse outcomes in critically ill patients.

## 5. Conclusions

Our results indicate that hematological parameter-driven inflammatory scores—particularly NLR and N/(LP)—are robust and accessible tools for predicting mortality in NPHL. However, the prognostic performance of these scores is dependent on the etiology of NPHL, which warrants consideration in clinical practice.

### Limitation

This study has some limitations. It was conducted in a single center and partially relied on retrospective data, which may limit generalizability. Inflammatory markers were only available at admission, and dynamic changes during hospitalization could not be assessed. The potential influence of comorbidities and treatments could not be fully adjusted for in the analysis. However, the large sample size (n = 545) strengthens the validity of our findings and mitigates the impact of these limitations.

## Figures and Tables

**Table 1:** Calculation formulas for hematological parameter-derived inflammatory scores.

Inflammatory Index	Abbreviation	Calculation Formula	Described By
Neutrophil-to-Lymphocyte Ratio	NLR	Neutrophil count/Lymphocyte count	Zahorec, 2001 [8]
Derived Neutrophil-to-Lymphocyte Ratio	dNLR	Neutrophil count/ (WBC count − Neutrophil count)	Proctor et al., 2012 [16]
Neutrophil-to-Lymphocyte and Platelet Ratio	N/(LP)	Neutrophil count/ (Lymphocyte count × Platelet count)	Koo et al., 2018 [17]
Platelet-to-Lymphocyte Ratio	PLR	Platelet count/Lymphocyte count	Turkmen et al., 2013 [18]
Lymphocyte-to-Monocyte Ratio	LMR	Lymphocyte count/Monocyte count	Merekoulias et al., 2010 [19]
Aggregate Index of Systemic Inflammation	AISI	(Neutrophil count × Platelet count × Monocyte count)/Lymphocyte count	Paliogiannis et al., 2018 [20]
Systemic Inflammation Index	SII	(Platelet count × Neutrophil count)/ Lymphocyte count	Hu et al., 2014 [21]
Systemic Inflammation Response Index	SIRI	(Neutrophil count × Monocyte count)/ Lymphocyte count	Qi et al., 2016 [22]

**Table 2:** Demographic, clinical, and laboratory characteristics and hematological parameter-derived inflammatory scores in patients with non-pancreatic hyperlipasemia (NPHL) according to etiology.

	Original (Non-COVID-19) Population		COVID-19 Population		
	NPHL Without Systemic Infection (n = 290)	NPHL with Bacterial Sepsis (n = 111)	*p* Value	NPHL with Viral Infection due to COVID-19 (n = 144)	*p* Value
**Demographic data**					
Gender (male) N (%)	154 (53.1)	67 (60.4)	0.217	81 (56.3)	0.541
Age (yr)	65 (53–76)	70 (57–76)	0.092	66 (55–76)	0.472
**Clinical and outcome data**					
Comorbidities, any N (%)	263 (90.7)	106 (95.5)	0.149	135 (93.7)	0.356
CCI	5 (3–8)	6 (4–9)	**0.001**	3 (2–5)	**<0.001**
Total length of hospitalization (d)	7 (2–16)	15 (8–27)	**<0.001**	11 (6–22)	**<0.001**
ICU (yes) N (%)	98 (33.8)	84 (75.7)	**<0.001**	69 (47.9)	**0.005**
ICU stay (d)	6 (3–15)	9 (3–16)	0.341	9 (5–15.5)	0.056
In-hospital mortality N (%)	40 (13.8)	50 (45.0)	**<0.001**	55 (38.2)	**<0.001**
**Biochemical parameters**					
Sodium (mmol/L)	139 (136–141)	140 (135–145)	**0.021**	137 (134–140)	**0.024**
Albumin (g/L)	37 (31–42)	29 (24–35)	**<0.001**	32 (28–36)	**<0.001**
BUN (mmol/L)	6.8 (4.6–13.6)	23 (10.7–37.2)	**<0.001**	10.7 (6.4–22.05)	**<0.001**
Creatinine (µmol/L)	78 (59–140)	218 (96–392)	**<0.001**	104 (72–186)	**<0.001**
Amylase level (U/L)	165 (110–229)	221 (132–344)	**<0.001**	167 (102–241)	0.798
Lipase (U/L)	266 (231–435)	425 (263–651)	**<0.001**	300.5 (243–556)	**<0.001**
CRP (mg/L)	21.1 (7.58–56.25)	114.6 (53.79–190.8)	**<0.001**	79.5 (19.3–141.3)	**<0.001**
PCT (ug/L) *	0.35 (0.13–0.91)	2.56 (1.08–6.32)	**<0.001**	0.28 (0.09–1.70)	0.187
**Blood count**					
WBC (G/L)	9.37 (7.23–12.8)	14.65 (10.11–21.46)	**<0.001**	11.20 (7.87–15.20)	**0.001**
Neutrophil granulocyte (G/L)	6.68 (4.80–10.00)	12.36 (8.96–18.03)	**<0.001**	9.05 (5.85–12.81)	**<0.001**
Lymphocyte count (G/L)	1.42 (0.95–2.01)	0.95 (0.62–1.54)	**<0.001**	1.11 (0.68–1.60)	**<0.001**
Monocyte count (G/L)	0.67 (0.48–1.01)	0.87 (0.59–1.29)	**0.001**	0.75 (0.49–1.08)	0.488
Platelet count (G/L)	236 (178–301)	219 (132–351)	0.224	235 (165–325)	0.980
**Hematological parameter-derived inflammatory scores**					
NLR	4.83 (2.69–8.78)	12.87 (7.81–21.75)	**<0.001**	7.35 (4.50–16.00)	**<0.001**
dNLR	2.74 (1.76–4.64)	6.13 (3.89–9.45)	**<0.001**	4.53 (2.95–8.02)	**<0.001**
N/(LP)	0.02 (0.01–0.05)	0.06 (0.03–0.14)	**<0.001**	0.03 (0.02–0.08)	**<0.001**
PLR	162.09 (110.93–262.45)	223.26 (115.57–396.68)	**0.009**	217.07 (125.16–355.1)	**<0.001**
LMR	1.95 (1.19–3.23)	1.13 (0.74–1.76)	**<0.001**	1.56 (1.03–2.34)	**<0.001**
AISI	765.40 (293.51–1862.99)	2588.8 (900.93–4690.27)	**<0.001**	1654.20 (481.20–4668.50)	**<0.001**
SII	1137.98 (540.18–2182.47)	2830.07 (1395.27–4921.08)	**<0.001**	1563.00 (809.40–4718.10)	**<0.001**
SIRI	3.34 (1.6–7.46)	11.96 (6.18–23.21)	**<0.001**	5.11 (2.80–11.94)	**<0.001**

Data are presented as median (interquartile range) for continuous variables and as number (percentage) for categorical variables. *p*-values refer to comparisons between the group with NPHL without systemic infection and the other two groups (NPHL with severe bacterial infection and COVID-19-associated NPHL). Statistically significant *p* values are indicated in bold. NPHL: non-pancreatic hyperlipasemia; CCI: Charlson Comorbidity Index; ICU: intensive care unit; BUN: blood urea nitrogen; CRP: C-reactive protein; PCT: procalcitonin; WBC: white blood cell; NLR: neutrophil-to-lymphocyte ratio; N/(LP): neutrophil-to lymphocyte and platelet ratio; PLR: platelet-to-lymphocyte ratio; LMR: lymphocyte-to-monocyte ratio; AISI: aggregate index of systemic inflammation; SII: systemic inflammation index; SIRI: systemic inflammation response index. * PCT was available in 274 cases in the non-COVID-19 population and 125 cases in the COVID-19 group.

**Table 3:** Predictive performance of hematological parameter-derived inflammatory scores for in-hospital mortality in the original non-COVID-19 population (n = 401).

Original Non-COVID-19	Survivors (n = 311)	Non-Survivors (n = 90)	*p* Value	AUROC	95% CI	Cut-Off	Sensitivity	Specificity
NLR	5.24 (2.84–9.66)	12.64 (6.64–19.50)	**<0.001**	0.747	0.691–0.803	>10.37	65.17	78.64
dNLR	2.96 (1.81–5.01)	5.84 (3.52–8.48)	**<0.001**	0.737	0.680–0.793	>4.19	70.79	66.67
N/(LP)	0.02 (0.01–0.05)	0.08 (0.03–0.14)	**<0.001**	0.772	0.717–0.827	>0.03	78.65	64.72
PLR	168.86 (112.86–285.31)	177.73 (102.76–326.78)	0.605	0.518	0.446–0.590	–	–	–
LMR	1.88 (1.15–3.1)	1.12 (0.69–1.84)	**<0.001**	0.664	0.598–0.730	≤1.78	75.28	54.05
AISI	887.4 (351.01–2189.47)	2148.92 (626.93–4531.25)	**<0.001**	0.630	0.561–0.699	>2330.97	49.44	76.38
SII	1224.78 (624.06–2561.27)	2294.76 (1010.94–4406.14)	**<0.001**	0.650	0.584–0.715	>1662.25	66.29	65.37
SIRI	3.81 (1.84–8.69)	11.47 (5.52–20.39)	**<0.001**	0.722	0.660–0.784	>5.94	74.16	64.08

Data are presented as median (interquartile range). The best discriminatory cut-off value and associated sensitivity and specificity values are not indicated for PLR since it did not reach statistical significance. Statistically significant *p* values are indicated in bold. AUROC: area under the receiver operating characteristic curve; NLR: neutrophil-to-lymphocyte ratio; dNLR: derived neutrophil-to-lymphocyte ratio; N/(LP): neutrophil-to lymphocyte and platelet ratio; PLR: platelet-to-lymphocyte ratio; LMR: lymphocyte-to monocyte ratio; AISI: aggregate index of systemic inflammation; SII: systemic inflammation index; SIRI: systemic inflammation response index.

**Table 4:** Predictive value of inflammatory markers based on univariable logistic regression in the original non-COVID-19 patient population (n = 401).

All Original	OR	CI	*p*
NLR	6.889	4.12–11.517	**<0.001**
dNLR	4.846	2.897–8.106	**<0.001**
N/(LP)	6.480	3.711–11.317	**<0.001**
LMR	3.582	2.106–6.091	**<0.001**
AISI	3.161	1.934–5.168	**<0.001**
SII	3.713	2.256–6.110	**<0.001**
SIRI	5.119	3.018–8.683	**<0.001**

This table presents the results of univariable logistic regression analyses evaluating the association between inflammatory markers and in-hospital mortality. Variables were analyzed as binary variables based on optimal cut-off values derived from ROC analysis. Statistically significant *p* values are indicated in bold. PLR was not included in this test due to lack of discriminative ability in ROC analysis. OR: odds ratio; CI: confidence interval. NLR: neutrophil-to-lymphocyte ratio; dNLR: derived neutrophil-to-lymphocyte ratio; N/(LP): neutrophil-to lymphocyte and platelet ratio; PLR: platelet-to-lymphocyte ratio; LMR: lymphocyte-to monocyte ratio; AISI: aggregate index of systemic inflammation; SII: systemic inflammation index; SIRI: systemic inflammation response index.

**Table 5:** Multivariable logistic regression of clinical and laboratory variables including N/(LP) in the original non-COVID-19 patient population (n = 401).

Original Non-COVID-19	OR	CI	*p*
Age	1.026	1.006–1.047	**0.010**
Sepsis	2.264	1.223–4.191	**0.009**
N/(LP) (>0.03)	3.980	2.039–7.768	**<0.001**
Amylase (>244 U/L)	2.496	1.345–4.634	**0.004**
Albumin (≤34 g/L)	3.166	1.669–6.007	**<0.001**
Constant	0.006		**<0.001**

This table presents the results of a multivariable logistic regression analysis including age and sepsis as well as amylase, albumin, and N/(LP) as dichotomized variables. The model was adapted from our previous work by substituting NLR with N/(LP). Statistically significant *p* values are indicated in bold. OR = odds ratio; CI = confidence interval; *p* values indicate statistical significance. NLR: neutrophil-to-lymphocyte ratio; N/(LP): neutrophil-to-lymphocyte and platelet ratio.

**Table 6:** Predictive performance of hematological parameter-derived inflammatory scores for in-hospital mortality in NPHL patients without systemic infection (n = 290).

No Systemic Infection	Survivors (n = 250)	Non-Survivors (n = 40)	AUROC	95% CI	*p*	Cut-Off	Sensitivity	Specificity
NLR	4.15 (2.55–7.60)	10.83 (6.12–15.97)	0.7805	0.704–0.857	**<0.001**	>5.260	87.50	60.48
dNLR	2.54 (1.72–4.03)	5.06 (3.06–7.42)	0.7506	0.671–0.830	**<0.001**	>4.250	67.50	76.21
N/(LP)	0.02 (0.01–0.04)	0.07 (0.02–0.10)	0.773	0.695–0.851	**<0.001**	>0.045	60.00	80.24
PLR	159.80 (107.34–253.85)	191.87 (126.03–334.90)	0.5755	0.476–0.675	0.1257	–	–	–
LMR	2.03 (1.32–3.41)	1.13 (0.70–2.21)	0.6796	0.580–0.779	**0.0003**	≤1.150	42.50	84.27
AISI	701.02 (268.52–1684.63)	1550.82 (466.76–3516.04)	0.6476	0.553–0.742	**0.0027**	>2456.6	70.00	66.13
SII	1056.87 (501.27–1844.27)	2002.55 (1075.45–3771.16)	0.6836	0.593–0.774	**0.0002**	>1402.5	67.50	73.39
SIRI	2.87 (1.44–6.44)	8.03 (3.43–15.89)	0.7237	0.634–0.813	**<0.001**	>5.950	52.50	81.45

This table presents median biomarker levels and the prognostic performance of various inflammatory scores in patients without systemic infection. Discrimination was assessed using ROC curves. Data are presented as median (interquartile range) if not otherwise indicated. Statistically significant *p* values are indicated in bold. AUROC: area under the receiver operating characteristic curve; CI: confidence interval; NLR: neutrophil-to-lymphocyte ratio; dNLR: derived neutrophil-to-lymphocyte ratio; N/(LP): neutrophil-to lymphocyte and platelet ratio; PLR: platelet-to-lymphocyte ratio; LMR: lymphocyte-to monocyte ratio; AISI: aggregate index of systemic inflammation; SII: systemic inflammation index; SIRI: systemic inflammation response index.

**Table 7:** Predictive performance of hematological parameter-derived inflammatory scores for in-hospital mortality in NPHL patients with sepsis (n = 111).

Sepsis	Survivors (n = 61)	Non-Survivors (n = 50)	AUROC	95% CI	*p*	Cut-Off	Sensitivity	Specificity
NLR	11.38 (7.34–21.11)	14.25 (9.11–22.10)	0.568	0.406–0.677	0.219	–	–	–
dNLR	5.47 (3.91–8.57)	7.41 (3.84–10.30)	0.563	0.453–0.673	0.257	–	–	–
N/(LP)	0.046 (0.03–0.09)	0.09 (0.04–0.16)	0.653	0.549–0.757	**0.006**	>0.087	53.06	77.05
PLR	256.76 (130.76–406.80)	169.35 (89.37–318.20)	0.616	0.509–0.722	**0.038**	≤171.15	51.02	68.85
LMR	1.14 (0.74–1.90)	1.08 (0.67–1.75)	0.503	0.393–0.613	0.952	–	–	–
AISI	2893.73 (1008.98–4491.68)	2475.71 (663.18–5858.84)	0.518	0.405–0.631	0.748	–	–	–
SII	2824.00 (1434.89–5442.03)	2836.14 (940.57–4737.29)	0.530	0.420–0.641	0.586	–	–	–
SIRI	10.04 (5.50–20.72)	14.27 (6.45–27.37)	0.576	0.467–0.686	0.170	–	–	–

Comparison of inflammatory indices between survivors and non-survivors among NPHL patients with sepsis (n = 111). Data are expressed as median (interquartile range). Discriminatory performance was assessed using receiver operating characteristic (ROC) curve analysis. Statistically significant *p* values are indicated in bold. AUROC: area under the receiver operating characteristic curve; CI: confidence interval; NLR: neutrophil-to-lymphocyte ratio; dNLR: derived neutrophil-to-lymphocyte ratio; N/(LP): neutrophil-to lymphocyte and platelet ratio; PLR: platelet-to-lymphocyte ratio; LMR: lymphocyte-to monocyte ratio; AISI: aggregate index of systemic inflammation; SII: systemic inflammation index; SIRI: systemic inflammation response index.

**Table 8:** Performance of hematological parameter-derived inflammatory scores in predicting mortality in COVID-19-related NPHL (n = 144).

COVID-19	Survivors (n = 89)	Non-Survivors (n = 55)	AUROC	95% CI	*p*	Cut-Off	Sensitivity	Specificity
NLR	5.70 (3.50–8.70)	16.00 (8.80–26.05)	0.810	0.736–0.884	**<0.004**	>10.65	73.58	85.06
dNLR	3.71 (2.47–4.85)	7.99 (4.77–13.22)	0.789	0.709–0.870	**<0.002**	>5.78	73.58	82.76
N/(LP)	0.025 (0.01–0.05)	0.065 (0.03–0.12)	0.773	0.695–0.850	**<0.001**	>0.035	67.92	70.93
PLR	158.85 (112.93–265.28)	301.32 (211.29–482.86)	0.726	0.639–0.812	**<0.003**	>215.00	75.47	63.95
LMR	1.73 (1.19–2.36)	1.27 (0.76–2.16)	0.618	0.518–0.717	**0.020**	≤0.915	32.08	90.80
AISI	1863.55 (568.43–5806.4)	1078.6 (288.25–3444.3)	0.608	0.510–0.706	**0.033**	≤476.05	39.62	84.88
SII	1160.00 (663.63–2475.28)	4158.00 (1491.85–7672.85)	0.763	0.683–0.843	**<0.001**	>2585.75	64.15	76.74
SIRI	4.59 (2.61–9.5)	7.62 (3.74–22.12)	0.670	0.575–0.765	**<0.001**	>5.89	64.15	68.97

Comparison of inflammatory indices between survivors and non-survivors in COVID-19-associated NPHL patients (n = 144). Data are presented as median (interquartile range). Discriminatory performance was assessed by ROC curve analysis. Statistically significant *p* values are indicated in bold. AUROC: area under the receiver operating characteristic curve; CI: confidence interval; NLR: neutrophil-to-lymphocyte ratio; dNLR: derived neutrophil-to-lymphocyte ratio; N/(LP): neutrophil-to lymphocyte and platelet ratio; PLR: platelet-to-lymphocyte ratio; LMR: lymphocyte-to monocyte ratio; AISI: aggregate index of systemic inflammation; SII: systemic inflammation index; SIRI: systemic inflammation response index.

## Data Availability

The data presented in this study are available on request from the corresponding author. The data are not publicly available due to privacy restrictions.

## Supplementary Materials

The following supporting information can be downloaded at https://www.mdpi.com/article/10.3390/biomedicines13071719/s1, Table S1: Etiologic classification and causes of in-hospital mortality in non-pancreatic hyperlipasemia (NPHL) without systemic infection (n = 290).

## Abbreviations

The following abbreviations are used in this manuscript:

ACE-2	Angiotensin-converting enzyme 2
AISI	Aggregate index of systemic inflammation
AP	Acute pancreatitis
BUN	Blood urea nitrogen
CCI	Charlson Comorbidity Index
CRP	C-reactive protein
CT	Computed tomography
dNLR	Derived neutrophil-to-lymphocyte ratio
ICU	Intensive care unit
LMR	Lymphocyte-to-monocyte ratio
MCV	Mean corpuscular volume
MRI	Magnetic resonance imaging
N/(LP)	Neutrophil-to-(lymphocyte × platelet) ratio
NLR	Neutrophil-to-lymphocyte ratio
NPHL	Non-pancreatic hyperlipasemia
PCT	Procalcitonin
PLR	Platelet-to-lymphocyte ratio
RDW	Red cell distribution width
SII	Systemic immune inflammation index
SIRI	Systemic inflammation response index
SOFA	Sequential Organ Failure Assessment
ULN	Upper limit of normal
WBC	White blood cell
WHO	World Health Organization
