# Supplementary material for: Hematological Parameter-Derived Inflammatory Scores in Non-Pancreatic Hyperlipasemia (NPHL)—The Prognosis Lies in the Blood
Source: Biomedicines. 2025 Jul 14;13(7):1719. doi: 10.3390/biomedicines13071719 (PMC12292475; doi:10.3390/biomedicines13071719)
Supplement: Supplementary file 1 [file biomedicines-13-01719-s001.zip › biomedicines-3705131-supplementary.pdf]

Supplementary Table S1

Etiologic classification and causes of in-hospital mortality in non-pancreatic hyperlipasemia (NPHL) without systemic infection (n = 290)

Etiology	Number of patients (n) total n=290	Number of deaths (n) total n=40	% of all deaths (n=40)
**Peptic ulcer**	2	1	2.5
**GI bleeding**	4	1	2.5
**Bowel Necrosis/perforation**	4	1	2.5
**Bowel obstruction**	8	1	2.5
**IBD**	5	0	0.0
**Cholecystitis**	8	0	0.0
**Cholangitis**	13	0	0.0
**Liver disease**	14	3	7.5
**Post-ERCP lipasemia**	16	0	0.0
**Pancreatic cancers**	22	2	5.0
**Non-pancreatic cancers**	27	4	10.0
**Haematological malignancies**	6	0	0.0
**Type 2 diabetes mellitus**	19	0	0.0
**Diabetic ketoacidosis**	6	0	0.0
**Neurosurgical pathology**	16	4	10.0
**Cardiovascular disease**	17	5	12.5
**Drugs side effect**	17	2	5.0
**Pregnancy-related conditions**	2	0	0.0

**Autoimmune disorders**	2	0	0.0
**Renal graft dysfunction**	1	0	0.0
**Infection without sepsis**	30	0	0.0
**Acute Kidney Injury**	51	16	40.0

This table presents the underlying conditions and causes of death in patients with non-pancreatic hyperlipasemia (NPHL) without evidence of systemic infection (sepsis or COVID-19). All patients had elevated serum lipase level (> 210 U/L). Most in-hospital deaths in this cohort were attributable to cardiovascular or respiratory failure, or terminal-stage liver insufficiency
GI: Gastrointestinal, IBD: Inflammatory bowel disease, ERCP: Endoscopic retrograde cholangiopancreatography.
